# Downregulation of Rap1GAP Expression Activates the TGF-*β*/Smad3 Pathway to Inhibit the Expression of Sodium/Iodine Transporter in Papillary Thyroid Carcinoma Cells

**DOI:** 10.1155/2021/6840642

**Published:** 2021-11-18

**Authors:** Zheng Yan, Wang Yangyanqiu, Han Shuwen, Mao Jing, Liao Haihong, Chen Gong, Jin Yin, Zhou Qing, Gao Weili

**Affiliations:** ^1^Department of Pathology, Affiliated Huzhou Hospital Zhejiang University, Affiliated Central Hospital Huzhou University, No. 1558, Sanhuan North Road, Wuxing District, Huzhou, Zhejiang Province, China 313000; ^2^Graduate of Affiliated Huzhou Hospital Zhejiang University, Affiliated Central Hospital HuZhou University, No. 1558, Sanhuan North Road, Wuxing District, Huzhou, Zhejiang Province, China 313000; ^3^Department of Oncology, Affiliated Huzhou Hospital Zhejiang University, Affiliated Central Hospital Huzhou University, No. 1558, Sanhuan North Road, Wuxing District, Huzhou, Zhejiang Province, China; ^4^Department of Laboratory Medicine, Huzhou Central Hospital, Affiliated Central Hospital HuZhou University, No. 1558, Sanhuan North Road, Wuxing District, Huzhou, Zhejiang Province, China 313000; ^5^Department of Nursing, Huzhou Central Hospital, Affiliated Central Hospital Huzhou University, No. 1558, Sanhuan North Road, Wuxing District, Huzhou, Zhejiang Province, China 313000; ^6^Department of Thyroid Surgery, Huzhou Central Hospital, Affiliated Central Hospital Huzhou University, No. 1558, Sanhuan North Road, Wuxing District, Huzhou, Zhejiang Province, China 313000; ^7^Undergraduate School of Clinic Medicine, Huzhou University, No. 1 Bachelor Road, Huzhou, Zhejiang Province, China 313000

## Abstract

**Objective:**

Rap1GAP is considered a tumor suppressor gene, but its regulatory mechanism in papillary thyroid cancer (PTC) has not been clearly elucidated. The aim of this study was to explore whether the regulation between Rap1GAP and sodium/iodine transporter (NIS) in tumorigenesis of PTC is mediated by TGF-*β*1.

**Methods:**

Western blotting (WB) and quantitative reverse-transcription polymerase chain reaction were performed to analyze the relationships between TGF-*β*1 concentration and NIS expression. After transfecting BCPAP cells with siRNAs, the Rap1GAP interference model was successfully established. Then, the expression and nuclear localization of TGF-*β*1 and pathway-related proteins were detected. Flow cytometry was applied to analyze cell apoptosis and cycle. WB was performed to detect apoptotic-related proteins. Wound healing and transwell assays were used to measure cell migration and invasion. EDU was performed to detect cell proliferative activity.

**Results:**

The results suggested that TGF-*β*1 could significantly inhibit the expression of NIS in both mRNA and protein levels. In BCPAP cells transfected with siRNA-Rap1GAP, the expression levels of TGF-*β*1, Foxp3, and p-Smad3 were significantly increased. By applying immunofluorescence assay, the nuclear localizations of T*β*R-1 and p-Smad3 were found to be activated. Moreover, anti-TGF-*β*1 can reverse the decrease in NIS expression caused by downregulation of Rap1GAP. Additionally, the knockdown of Rap1GAP could alter the cell apoptosis, cycle, migration, invasion, and proliferation of BCPAP.

**Conclusion:**

The downregulation of Rap1GAP expression can activate the TGF-*β*/Smad3 pathway to inhibit NIS expression and alter the tumor cell functions of PTC.

## 1. Introduction

Papillary thyroid cancer (PTC) is the most common histological type of differentiated thyroid malignancies, accounting for about 85% of all pathologic types [1, 2]. The extensive application of ultrasound screening and ultrasound-guided fine needle biopsy has promoted the detection and diagnosis of PTC [3, 4]. Currently, treatments for PTC include surgery, thyroid hormone suppression, iodine-131 therapy, and adjuvant radiation therapy [5]. Although PTC is a low-grade malignancy with a good prognosis, there are still approximately 10-15% of cases showing tumor heterogeneity and aggressive variation along with unique clinical, pathological, and molecular characteristics [6]. These histological variations are associated with tumor recurrence, metastasis, therapeutic resistance, and radioiodine resistance and may eventually lead to lower survival rates [7]. Large-scale genomic characterization studies of PTC have revealed the key role of genetic alterations in the oncogenesis of this disease [8]. In particular, BRAFV600E and RAS are believed to be major signaling drivers, and about 50% of PTC cases carry BRAFV600E mutations [9]. However, the cancerization of PTC is a complex biological process characterized by a variety of molecular abnormalities. Therefore, decoding the molecular mechanisms related to the pathogenesis of PTC may help to identify new therapeutic targets.

Rap1 GTPase-activating protein (Rap1GAP) locates on chromosome 1p36.1-p35 and encodes a protein with the molecular weight of 73 kDa [10]. Rap1GAP regulates the specific GAP activity for Rap1 and is known as a tumor suppressor gene and plays a key role in human tumor progression including thyroid cancer [11]. Previous studies have found the deficiency expression of Rap1GAP in PTC, and the deletion of Rap1GAP allele was detected in about 20% of PTC cases [12, 13]. Studies on the upstream regulatory mechanisms revealed that miR-3121-3p could mediate the expression of Rap1GAP and affect the proliferation and metastasis of PTC cells by regulating the MAPK signaling pathway [14]. However, limited reports failed to fully explain the regulatory mechanism of Rap1GAP in PTC.

Sodium iodide symporter (NIS), a transmembrane glycoprotein, mediates the movement of active iodine in the thyroid gland and other tissues, and the expression of NIS can be selectively enhanced in thyroid cells by thyroid-stimulating hormone (TSH) [15, 16]. It has been reported that BRAFV600E mutation in PTC is related to expression changes of Rap1GAP, while the inhibition of BRAFV600E mutation can restore the expression of NIS in PTC cells, thereby illustrating the potential regulatory relationships between Rap1GAP and NIS in PTC development [13, 17]. Notably, the expression of NIS and its targeting localization on the plasma membrane of thyroid cells are not only regulated by TSH and iodine but also closely related to the concentration of transforming growth factor beta1 (TGF-*β*1) [18]. It has been shown that NIS expression level is decreased in tumor cells treated with TGF-*β*1 and NIS may be regulated by TGF-*β*1 [19, 20]. However, whether the regulation mechanism of Rap1GAP on NIS in PTC is mediated by TGF-*β*1 and related signaling pathway has not been elucidated.

Combined with the above findings, this study speculated that Rap1GAP regulates the expression of NIS by mediating TGF-*β*1 and its signaling pathway in PTC. To further investigate the hypothesis, we experimentally explored the expression relationship between Rap1Gap, TGF-*β*1, and NIS in PTC cells. Subsequently, siRNAs were applied to silence the expression of Rap1GAP, and functional changes of transfected PTC cells including the cell apoptosis, cell cycle, migration, and invasion, as well as expression and localization changes of TGF-*β*1 and TGF-*β*/Smad3 pathway proteins, were detected. Our study proposed a new regulatory axis of Rap1GAP in inhibiting PTC progression and provided theoretical basis to discovery novel therapeutic targets for disease.

## 2. Methods

### 2.1. Cell Lines and Cell Culture

BCPAP cells provided by KeyGen BioTECH Co., Ltd. (Nanjing, China), were human thyroid carcinoma papillary cells. They were isolated from a 76-year-old woman with metastatic papillary thyroid tumor in 1992. Based on their growth characteristics, they were cultured in a 90% RPMI1640 culture medium supplemented with 10% fetal bovine serum (FBS). The cells were then grown in an incubator with saturated humidity and 5% CO_2_ at 37°C. They usually grow into adherent cells, and their shapes are usually fusiform or round in shape. Cells in a logarithmic growth phase were extracted for subsequent experiments.

### 2.2. Quantitative Reverse-Transcription Polymerase Chain Reaction (qRT-PCR) Analysis

The total RNA was extracted from BCPAP using the TRIzol (15596-026, Invitrogen, USA). After reverse transcriptional reaction, cDNA was quantified by the Step one plus Real time-PCR system (ABI, USA) by following the standard procedures of One Step TB Green™ PrimeScript™ RT-PCR Kit II (RR086B, TaKaRa, Japan). The primers were designed by Primer6, synthesized by KeyGen BioTECH Co., Ltd., and purified by polyacrylamide gel electrophoresis (PAGE). The gene accession number and sequences of primers used in the present study are shown in Table 1. The mRNA expression level was normalized against GAPDH.

### 2.3. Western Blotting (WB) Analysis

The total protein was obtained using extraction kit (KGP250, KeyGen BioTECH, Nanjing, China), followed by the quantification using BCA protein concentration detection kit (KGA902, KeyGen BioTECH, Nanjing, China). By applying gel preparation kit (KGP113, KeyGen BioTECH, Nanjing, China), sodium dodecyl sulfate- (SDS-) PAGE was conducted followed by the membrane transfer. Thereafter, WB was performed, using anti-NIS (24324-1-AP, Proteintech Group, Inc., China), anti-Rap1GAP (ab32373, Abcam, UK), anti-TGF-*β*1 (ab215715, Abcam, UK), anti-Foxp3 (bs10211R, Bioss, China), anti-T*β*R1 (bs0638R, Bioss, China), anti-p-Smad3 (ab52903, Abcam, UK), anti-Smad3 (ab40854, Abcam, UK), anti-bcl-2 (ab182858, Abcam, UK), and anti-Bax (ab182733, Abcam, UK) with dilution rates of 1 : 1000, 1 : 10000, 1 : 1000, 1 : 1000, 1 : 1000, 1 : 2000, 1 : 1000, 1 : 2000, and 1 : 2000, respectively. After incubation with the secondary antibody, the membrane was colorized and imaged using G: BOX chemiXR5 (syngene, UK).

### 2.4. Retroviral Construction Infection and Transfection

To downregulate the expression of Rap1GAP, three siRNA plasmids synthesized by KeyGen BioTECH Co., Ltd. (Nanjing, China), were transfected into BCPAP using Lipofectamine 3000 (L3000015, Invitrogen, USA). The sequences were listed as follows: siRNA-Rap1GAP-1-F—GCUACAAGGCAGAGAAGUUTT; siRNA-Rap1GAP-1-R—AACUUCUCUGCCUUGUAGCTT; siRNA-Rap1GAP-2-F—AGGUGAAGCUCGAGUGCAATT; siRNA-Rap1GAP-2-R: UUGCACUCGAGCUUCACCUTT; siRNA-Rap1GAP-3-F—GCAAGGAGCAUUUCAAUUATT; and siRNA-Rap1GAP-3-R—UAAUUGAAAUGCUCCUUGCTT. After 24 and 48 hours of transfection, qRT-PCR was used to verify the expression of Rap1GAP (compared with the blank and negative control (NC) groups). These verified cells were used for further experiments.

### 2.5. Enzyme-Linked Immunosorbent Assay (ELISA)

BCPAP cells were cultivated at an appropriate density, and a cell culture supernatant was collected for ELISA with references to the instructions of human TGF-*β*1 ELISA kit (KGEHC107b, KeyGen BioTECH, Nanjing, China). Then, the absorbance of each sample was measured by a microplate reader (SpectraMax M3, MD, USA), and the concentration of TGF-*β*1 was calculated according to the standard curve.

### 2.6. Immunofluorescence Analysis

After drying naturally, BCPAP cells were immersed in 4% paraformaldehyde fixation solution to improve the cell permeability. Then, BCPAP was coincubated with anti-T*β*R1 (bs0638R, Bioss, China) and anti-p-Smad3 (ab52903, Abcam, UK) with a dilution rate of 1 : 100 for 2 hours. Followed by exposing to fluorescent dye-conjugated secondary antibody (TRITC) diluted at 1 : 100, BCPAP was mounted with DAPI. The expression of target protein was observed and recorded by a laser scanning confocal microscope (710, Zeiss, Germany).

### 2.7. Cell Apoptosis Assay

A flow cytometer (FACSCalibur, Becton-Dickinson, USA) was employed for cell apoptosis assay using Annexin V-APC/7-AAD Apoptosis Assays Kit (KGA1024, KeyGen BioTECH, Nanjing, China). For cell cycle analysis, a PI single staining method was implemented followed by the flow cytometry analysis using Cell Cycle Assay Kit (KGA511, KeyGen BioTECH, Nanjing, China).

### 2.8. Cell Migration and Invasion Assay

Cell migration was measured by wound healing assay, while cell invasion was detected by transwell assay. BCPAP cells at the logarithmic growth phase were digested and inoculated into a six-well plate (3516, Corning Incorporated, USA). For invasion assay, cells were seeded into the upper chamber with or without Matrigel (356234, BD, USA) after 48 h transfection, and the lower chamber was placed into culture medium with 20% FBS. Followed by 24 h incubation, transwell was removed and cells were dried for staining with crystal violet (C3886, Sigma, USA). Then, cells were recorded with a magnification of 200x and finally counted. For migration assay, BCPAP cells were transfected with lentivirus and a negative control group was set. Subsequently, cultured cells were evenly lined with a sterile spear tip and continued to grow for 24 hours. Finally, cells were photographed under a 100x magnification microscope (IX51, OLYMPUS, Japan), and the migration distance was measured.

### 2.9. Cell Proliferation Assay

Cell proliferation was detected by EDU assay. The cell suspension was added to the 96-well cell culture plate and cultured in a 5% CO_2_ incubator at 37°C for 24 h and cleaned twice with PBS. Edu culture was added, then discarded, and cleaned twice. Each well was treated with cell fixative solution, glycine, PBS, 1×Apollo® staining reaction solution, osmotic agent (0.5% TRiton X-100 PBS), and 1×Hoechst 33342 reaction solution and monitored by high content cell imaging system (MD).

### 2.10. Animal Assay

Ten 4-week-old female BALB/C nude mice (SCXK (Shanghai) 2017-0005) were provided by Shanghai Slack Laboratory Animal Co., Ltd. Animals were fed in the room temperature of 20~26°C (diurnal temperature variation ≤ 4°C), relative humidity of 40~70%, 12 hours of an alternating light and dark (7 o'clock lights on, 19 o'clock lights off) environment. The mice were randomly divided into two groups. The cell suspension concentration of BCPAP cells and BCPAP cells that downregulated the expression of Rap1GAP was 1 × 10^7^/mL, and 0.1 mL of each cell suspension was inoculated into the left thyroid lobe of nude mice of the two groups. Two weeks later, the nude mice were sacrificed. The tumor was surgically removed and weighed. An oil gauge caliper was used to measure tumor diameter.

### 2.11. Statistical Analysis

GEL-PRO32 software was used to analyze the gray scale of WB results. All data was expressed as means ± standard deviation (SD) of at least triplicate and was processed by GraphPad Prism 5 (GraphPad, USA). Differences between two groups were analyzed by a *t*-test. Two-tailed *p* < 0.05 was set as statistical significance.

## 3. Result

### 3.1. TGF-*β*1 Inhibits the Expression of NIS in BCPAP

**Figure 1:**
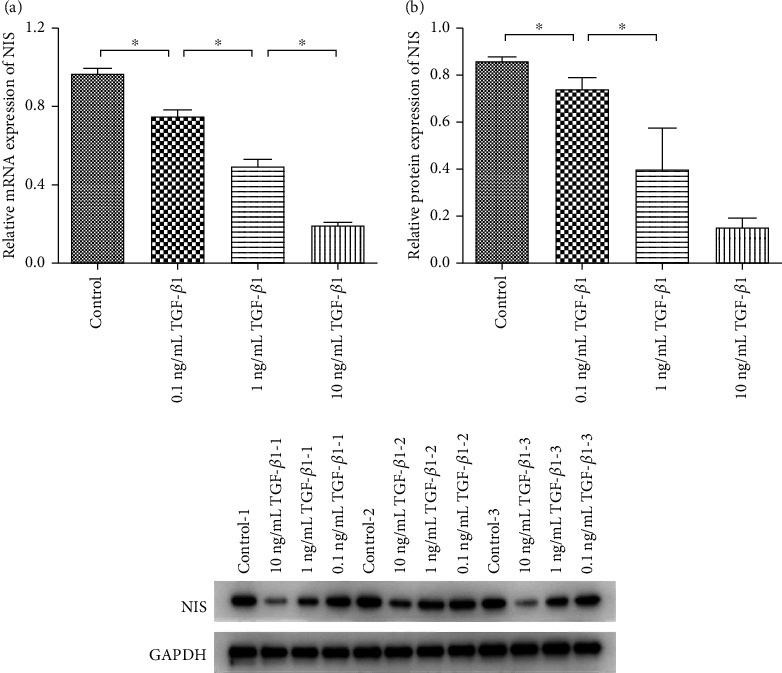
The expression of NIS detected by qRT-PCR (a) and WB (b) (^∗^*p* < 0.05 compared between two groups). Independent experiments were conducted in triplicates.

To explore whether the expression level of NIS was affected by TGF-*β*1 in PTC, qRT-PCR and WB were carried out on BCPAP. Cells were grouped as BCPAP, BCPAP + 0.1 ng/mL TGF-*β*1, BCPAP + 1 ng/mL TGF-*β*1, and BCPAP + 10 ng/mL TGF-*β*1, and the experiment was set up with three replicates in each groups. The results (Figures 1(a) and 1(b)) suggested that the expressions of NIS in both mRNA and protein levels were significantly decreased with the increase of TGF-*β*1 concentration (*p* < 0.05). This finding suggested that TGF-*β*1 could inhibit the expression of NIS in BCPAP.

### 3.2. Downregulation of Rap1GAP Activates TGF-*β*/Smad3 Pathway in BCPAP

**Figure 2:**
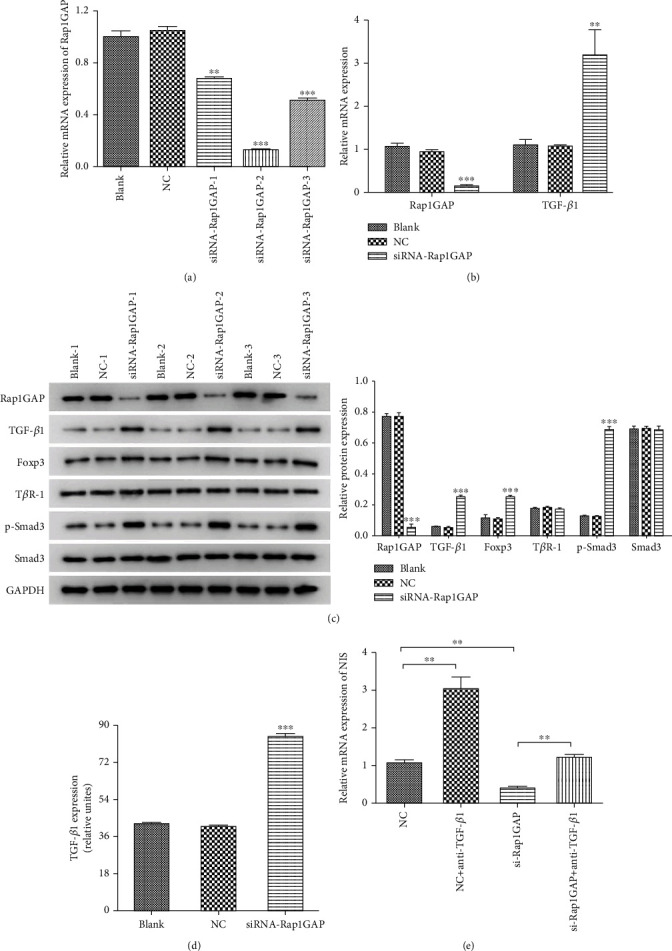
Expression changes in TGF-*β*/Smad3 pathways were detected by qRT-PCR, WB, and ELISA with knockdown of Rap1GAP. (a) The knockdown efficiencies of three Rap1GAP-siRNAs were detected by qRT-PCR. (b) The mRNA expression of TGF-*β*1 in the siRNA-Rap1GAP group. (c) Protein expression in TGF-*β*/Smad3 pathway was detected by WB. (d) The protein expression of TGF-*β*1 in siRNA-Rap1GAP group was detected by ELISA. (e) Anti-TGF-*β*1 can rescue the downregulation of NIS expression caused by siRNA-RAP1GAP. ^∗∗^*p* < 0.01 and ^∗∗∗^*p* < 0.001 compared with the blank and NC groups. Independent experiments were conducted in triplicates.

In order to investigate the regulatory relationship between Rap1GAP and TGF-*β*1, three siRNAs of Rap1GAP plasmid were constructed and then transfected into BCPAP. The results of qRT-PCR showed that siRNA-Rap1GAP-2 had the highest knockdown efficiencies among all three siRNAs with *p* < 0.001 (Figure 2(a)). With the downregulation of Rap1GAP, the mRNA expression of TGF-*β*1 was found to be significantly increased (Figure 2(b)). Therefore, we further detected the expression of key proteins involved in TGF-*β*/Smad3 pathways by WB. As shown in Figure 2(c), the knockdown of Rap1GAP can significantly increase the expression of TGF-*β*1, Foxp3 and p-Smad3 (*p* < 0.001). However, the expression levels of T*β*R-1 and Smad3 were not significantly changed. The increased expression of TGF-*β*1 in the siRNA-Rap1GAP group was also verified by ELISA (Figure 2(d), *p* < 0.001). Moreover, NIS was found to be downregulated and expressed in BCPAP infected with siRNA-Rap1GAP, which could be further reversed by anti-TGF-*β*1 (Figure 2(e), *p* < 0.01). The above findings illustrated that downregulation of Rap1GAP can activate the TGF-*β*/Smad3 pathway in BCPAP, and anti-TGF-*β*1 can rescue the downregulation of NIS expression caused by siRNA-Rap1GAP.

**Figure 3:**
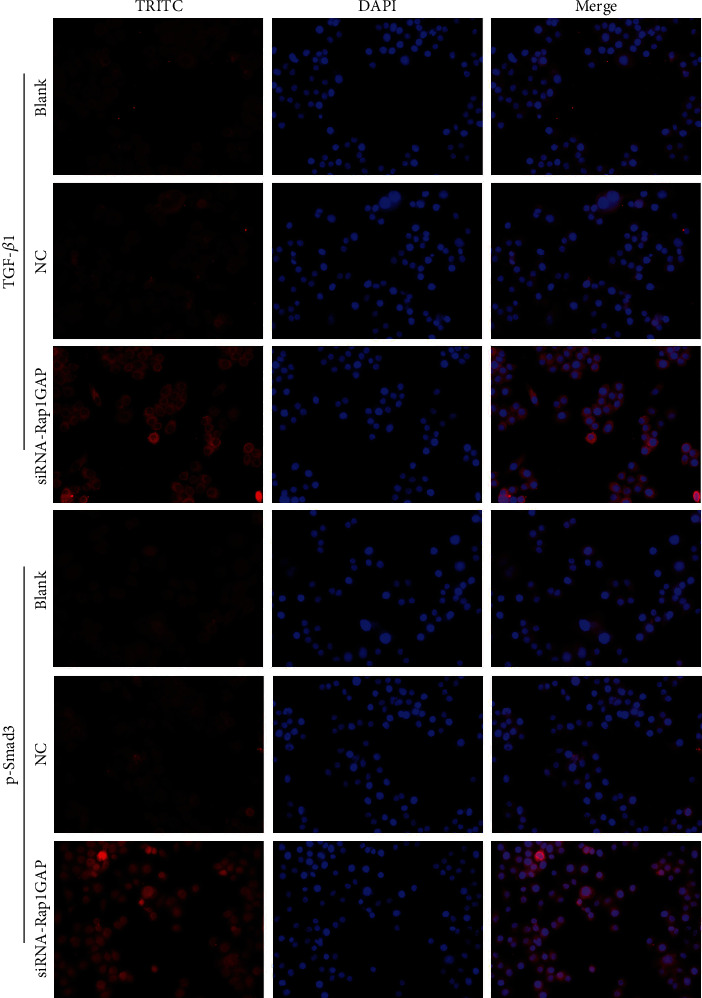
Immunofluorescence detection of TGF-*β*1 and p-Smad3 in transfected BCPAP.

We further detected the plasma membrane localization of TGF-*β*1 and p-Smad3 in BCPAP transfected with siRNA-Rap1GAP by immunofluorescence assay. As shown in Figure 3, membrane localizations of TGF-*β*1 and p-Smad3 were notably induced in the siRNA-Rap1GAP group.

### 3.3. The Deficiency Expression of Rap1GAP Affects Cell Apoptosis and Cycle

**Figure 4:**
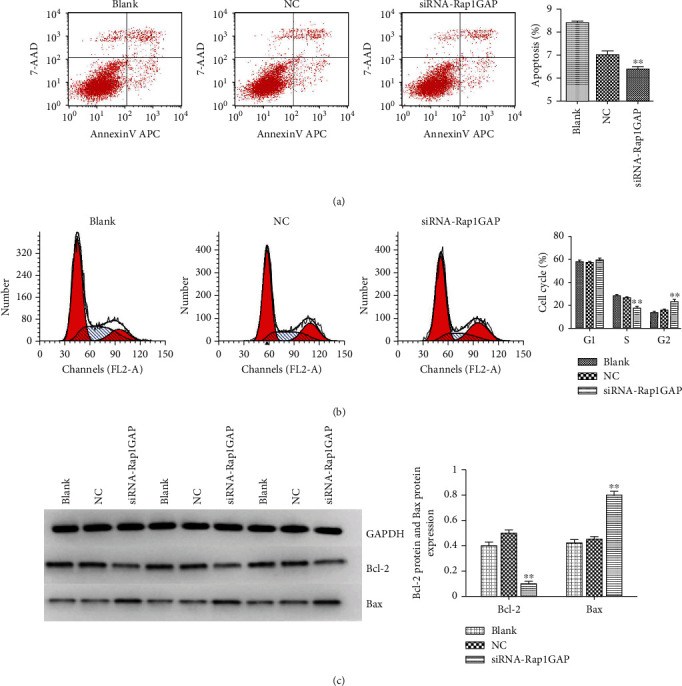
Flow cytometry detects cell apoptosis (a) and cycle (b) of transfected BCPAP. Western blot detects the expression levels of antiapoptotic protein (Bcl-2) and apoptotic promoting protein (Bax) (c) of transfected BCPAP. ^∗∗^*p* < 0.01 compared with the blank and NC groups. Independent experiments were conducted in triplicates.

Flow cytometry analysis was performed to study the effects of Rap1GAP on cell apoptosis (Figure 4(a)) and cycle (Figure 4(b)). The results suggested that the apoptosis rate of BCPAP in the siRNA-Rap1GAP group was significantly lower than those in the blank and NC groups (*p* < 0.01). Meanwhile, the low expression of Rap1GAP resulted in a significant increase of BCPAP cell numbers in G2 phase and a significant decrease of cell numbers in S phase, thereby illustrating a G2 phase retardation of transfected BCPAP. Western analysis was used for further detect the apoptosis-related proteins. The results showed that antiapoptotic protein (bcl-2) was downregulated and apoptotic promoting protein (Bax) was upregulated, compared with the blank and NC groups (*p* < 0.01).

### 3.4. Knockdown of Rap1GAP Enhances the Function of Tumor Cells and Promote Tumor Growth

**Figure 5:**
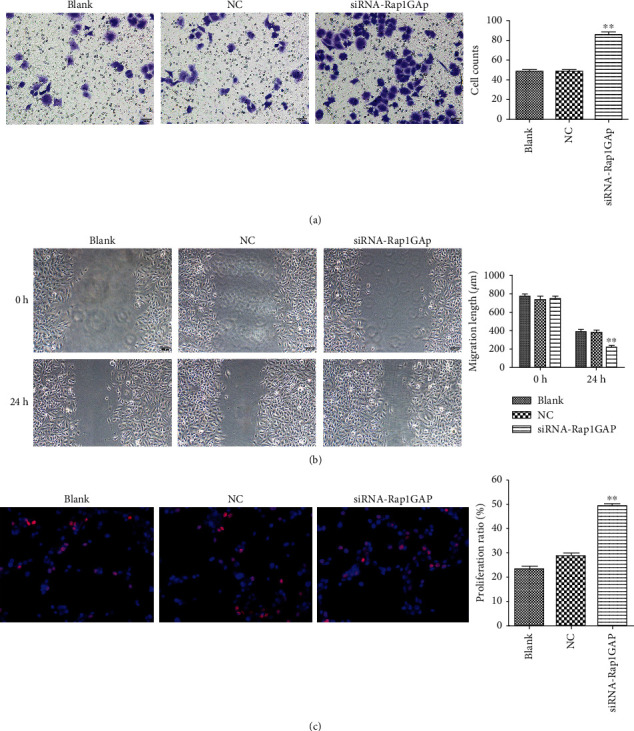
Cell invasion, migration, and proliferation of transfected BCPAP cells were measured by transwell (a), wound healing (b), and EDU assays (c), respectively. ^∗∗^*p* < 0.01 compared with the blank and NC groups. Independent experiments were conducted in triplicates.

**Figure 6:**
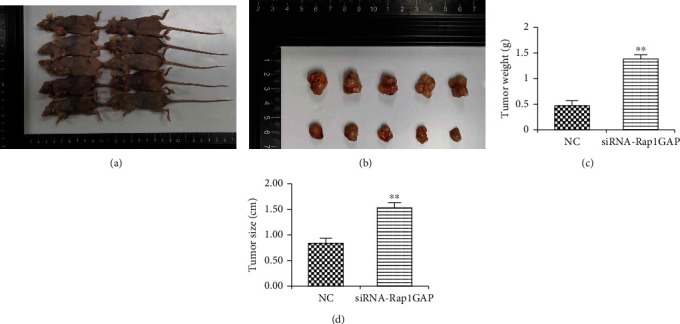
The mouse model of orthotopic transplanted tumor was constructed (a). Tumors removed from the mouse model (b). The weight (c) and size (d) of orthotopic transplantation tumor were measured. ^∗∗^*p* < 0.01 compared with the NC group.

Transwell, wound healing, and EDU assays were finally performed to explore cell invasion, migration, and proliferation, respectively. In BCPAP with low expression of Rap1GAP, the number of invaded cells was significantly increased (Figure 5(a)). Additionally, after 24 h culture, we found that the scratch distance of transfected cells was significantly reduced (Figure 5(b)) and the proliferation ration of transfected BCPAP was significantly increased (Figure 5(c)). What is more, the weight and size of orthotopic transplantation tumor in the siRNA-Rap1GAP group were increased (Figure 6). These findings suggested that knockdown of Rap1GAP can promote cell migration, invasion, and proliferation of BCPAP and promote tumor growth.

## 4. Discussion

Attributed to the improvement of diagnostic level, the number of confirmed cases of PTC has increased rapidly thereby leading to a steadily rising incidence of thyroid cancer all over the world [21]. Although PTC is an indolent tumor, papillary carcinoma with an aggressive variant is highly invasive and has a tendency to dedifferentiate and may contribute to the eventual development of poorly differentiated or undifferentiated thyroid carcinoma [22]. Distal metastasis is the dominant phenotype of invasive variants, and the median survival of patients with distant metastasis was estimated to be 4.1 years, while the 10-year specific survival rate dropped to 26% [23]. Several gene mutations have been shown to be specific in PTC, and combined application with needle biopsy can improve the accuracy of PTC diagnosis [9]. Therefore, a more comprehensive understanding of the molecular mechanisms of cell invasion and metastasis, as well as the identification of predictive PTC biomarkers, can improve the diagnostic efficiency of malignant tumors and reduce the prognostic risk.

Rap1GAP is a family member of GTPase-activating proteins and is believed to be involved in cancer progression [24]. It has been reported that the expression of Rap1GAP is abundant in well-differentiated rat thyroid cells, but it is selectively reduced in tumor cell lines with mesenchymal morphology, and restoration of Rap1GAP expression can inhibit tumor cell migration and invasion [12]. To further explore the effect of Rap1GAP on PTC, we downregulated the expression of Rap1GAP in BCPAP using siRNA plasmids and found that the cell migration and invasion were significantly promoted (*p* < 0.01). The depletion of Rap1GAP in thyroid tumors may enhance Src kinase activity, thereby promoting skeletal remodeling and motility in cancer cells [25], which may well explain our results. Tsygankova et al. elaborated that Rap1Gap-deficient tumor cells exhibited cell-cell adhesion defects and abnormal distribution of adhesion-junction proteins [26]. Furthermore, Rap1GAP has also been identified as a more effective cell-matrix inhibitor, and overexpression of Rap1GAP in cancer cells can impair cell proliferation and migration on type IV collagen [27]. Therefore, we hypothesized that low expression of Rap1GAP in PTC cells may promote tumor invasion by altering cell-cell and cell-matrix adhesion.

To investigate the correlation of Rap1GAP and NIS in PTC cells, we experimentally confirmed that Rap1GAP silence could induce the downregulation of NIS expression in BCPAP. BRAF may play an important role in this relationship, because it is believed that the presence of BRAF V600E mutation is associated with the deletion of Rap1GAP allele and changes in protein expression [13], while hiSTONE deacetylation of NIS promoter is the basis of BRAF V600E promoting NIS silencing in thyroid cancer [28]. Additionally, in a mouse model with PTC oncogene knock-in, decreased expression of the thyroid specific gene NIS and increased expression of the epithelial-mesenchymal transition regulator TGF-*β*1 were detected at the same time [29]. To learn whether Rap1GAP regulates NIS expression through TGF-*β*1, we firstly studied the expression relationship between them and confirmed that the expression of NIS was negatively associated with the TGF-*β*1 concentration in BCPAP cells. In BCPAP cells transfected with siRNA-Rap1GAP, we also observed that the expression and nuclear localization of TGF-*β*1 and TGF-*β*/Smad3 pathway-related proteins including Foxp3 and p-Smad3 were significantly activated. By constructing lentivirus-mediated Foxp3 overexpression cells, a related study revealed that upregulated Foxp3 in PTC cells may significantly reduce transcription and protein levels of NIS and also activate the TGF-*β*1 pathway [30]. Additionally, TGF-*β*1 may induce the downregulation of NIS gene expression in thyroid follicular cells by activating Smad3 [31]. Restoration experiments in this present study further confirmed that anti-TGF-*β*1 could rescue the downregulation of NIS expression caused by Rap1GAP deficiency.

This study explored the regulatory relations of Rap1GAP-TGF-*β*/Smad3-NIS in PTC cells but only did the experiment in one cell line. Additionally, there is still a lack of valuable animal experimental results to clarify this regulatory mechanism. More cell lines and animal experiments are needed to verify this mechanism. Based on the understanding of this regulatory relationship, we will further collect tissue samples from patients to explore the potential correlation between Rap1GAP expression and clinicopathological characteristics, so as to excavate the clinical value of Rap1GAP as a molecular marker for diagnosis and prognosis of patients with PTC.

## 5. Conclusion

In this present study, we found that TGF-*β*1 inhibited NIS expression and anti-TGF-*β*1 reversed the decrease in NIS expression caused by downregulation of Rap1GAP. Meanwhile, the knockdown of Rap1GAP can induce the activation of TGF-*β*1/Samd3 pathway and alter cell apoptosis, cycle, migration, and invasion in PTC cells. Our results provide a new perspective on the molecular regulatory mechanism of Rap1Gap in inhibiting PTC progression.

## Figures and Tables

**Table 1:** Gene accession number and sequences of primers.

Gene	Gene accession	Sequences of primers
NIS	NM_000453	Forward: CCTCTGCTGGTGCTGGACATCT Reverse: TGCTGGTGGATGCTGTGCTGAG
Rap1GAP	NM_001388273 XM_017001977	Forward: GGCGACGAGGACAAGATGGAGA Reverse: TGGCTGGTGGACACGGTGTT
TGF-*β*1	NM_000660	Forward: AGGACCTCGGCTGGAAGTGGAT Reverse: AGGACCTTGCTGTACTGCGTGT
GAPDH	NM_000453	Forward: CAAATTCCATGGCACCGTCA Reverse: AGCATCGCCCCACTTGATTT

## Abbreviations

PTC: Papillary thyroid cancer
Rap1GAP: Rap1 GTPase-activating protein
NIS: Sodium iodide symporter
TSH: Thyroid-stimulating hormone
TGF-*β*1: Transforming growth factor beta1
FBS: Fetal bovine serum
qRT-PCR: Quantitative reverse-transcription polymerase chain reaction
PAGE: Polyacrylamide gel electrophoresis
F: Forward
R: Reverse
WB: Western blotting
SDS-PAGE: Sodium dodecyl sulfate polyacrylamide gel electrophoresis
NC: Negative control
ELISA: Enzyme-linked immunosorbent assay
SD: Standard deviation.

## Additional Points

*Highlights*. (1) TGF-*β*1 may inhibit the expression of NIS in BCPAP cells. (2) The downregulation of Rap1GAP can significantly reduce the expression of NIS. (3) The knockdown of Rap1GAP alters cell apoptosis, cycle, migration, invasion, and proliferation in BCPAP. (4) Anti-TGF-*β*1 can reverse the decrease in NIS expression caused by downregulation of Rap1GAP
